# Pharmacogenetic associations of *CYP2D6* and *CYP2C19* variants with anticholinergic drug burden and hyposalivation

**DOI:** 10.3389/fphar.2025.1713345

**Published:** 2025-11-19

**Authors:** Olga A. Korczeniewska, Scott Diehl, Abdul Basir Barmak, Tong Tong Wu, Eli Eliav, Szilvia Arany

**Affiliations:** 1 Center for Orofacial and Temporomandibular Disorders, Department of Diagnostic Sciences, Rutgers School of Dental Medicine, Rutgers, The State University of New Jersey, Newark, NJ, United States; 2 Department of Dentistry, Eastman Institute for Oral Health, University of Rochester, Rochester, NY, United States; 3 Department of Biostatistics and Computational Biology, University of Rochester, Rochester, NY, United States; 4 Specialty Care, Eastman Institute for Oral Health, University of Rochester, Rochester, NY, United States

**Keywords:** pharmacogenetics, medications, saliva flow, CYP450, anticholinergic burden

## Abstract

**Introduction:**

Anticholinergic medications frequently cause hyposalivation (decreased saliva flow) through parasympathetic inhibition. This adverse effect is related to anticholinergic burden, reflecting the cumulative exposure to drugs with anticholinergic properties. Genetic variation in CYP genes, which encode drug-metabolizing enzymes, alters drug metabolism, potentially influencing systemic anticholinergic burden. This study investigated whether polymorphisms in the *CYP2D6* and *CYP2C19* genes are associated with anticholinergic burden and hyposalivation.

**Methods:**

Adults taking at least one CYP substrate anticholinergic medication reporting xerostomia (oral dryness) were recruited. Anticholinergic burden was quantified using the Anticholinergic Drug Scale (ADS) and Serum Anticholinergic Activity (SAA). Salivary assessment included unstimulated whole saliva (UWS) and minor saliva flow (MSF). Participants were genotyped for functional variants of *CYP2D6* and *CYP2C19*.

**Results:**

*CYP2D6* rs28371725 polymorphism was associated with low MSF and increased SAA in severe hyposalivation (genotype relative risk: 12.75, 95% CI 1.45–112.12). Additionally, variants (rs28371706, rs5030655) were associated with high ADS scores. Individuals with reduced CYP2D6 activity presented with higher systemic exposure and a greater anticholinergic burden for a given dose, as reflected by higher ADS and SAA. *CYP2C19* polymorphisms showed no strong associations with salivary outcomes or anticholinergic burden.

**Conclusion:**

Genetic variation in *CYP2D6* contributes to interindividual differences in systemic anticholinergic burden and hyposalivation. Pharmacogenetic profiling of CYP450 genes may help identify patients at risk of xerostomia from anticholinergic therapy, supporting more personalized and optimized anticholinergic prescribing.

## Introduction

Anticholinergic toxicity is a severe side effect of medications that block cholinergic receptors in the body (Tune et al., 1981). By inhibiting cholinergic signaling, these medications interfere with the normal function of the parasympathetic nervous system, leading to significant side effects across multiple organs (Soukup et al., 2017). Among these, xerostomia, or subjective oral dryness, often combined with hyposalivation resulting from decreased saliva secretion, is the most frequently reported side effect, as the salivary glands are the most sensitive organs to parasympathetic inhibition (Desoutter et al., 2012; Singh and Papas, 2014). Decreased salivary secretion can lead to difficulties in chewing and swallowing, a higher risk of dental caries and periodontal disease, diminished quality of life, oral pain, mucositis, and increased susceptibility to infections (Arany et al., 2021). Previous studies have shown that the use of various anticholinergic medications, such as antidepressants and urinary antispasmodics, can increase the risk of hyposalivation (Leung et al., 2002; Ness et al., 2006). Anticholinergic medications contribute to elevated anticholinergic burden, which may be estimated by scoring medications according to their antagonistic potential on parasympathetic signaling. The anticholinergic drug scale (Khatri et al., 2022), which quantifies the anticholinergic burden of medications, has been associated with xerostomia and decreased saliva secretion (Carnahan et al., 2006; Sevilla-Sanchez et al., 2018). The “gold standard” method, serum anticholinergic activity (SAA), measures anticholinergic burden directly from blood through a radioligand assay that measures the *in vitro* anticholinergic activity of compounds (Hachisu et al., 2015; Nobrega et al., 2017). Accordingly, higher ADS scores were found to be reflected in higher SAA (Hori et al., 2014; Kersten et al., 2013; Lampela et al., 2017).

While pharmacological mitigation, such as dose adjustment, substitution with medications of lower anticholinergic activity, or discontinuation of non-essential medications, is a cornerstone of managing medication-induced hyposalivation, clinical experience shows that these strategies do not fully account for individual variability in salivary outcomes (Miranda-Rius et al., 2015; Ostrowska et al., 2025). Prior work has outlined a structured clinical framework that emphasizes medication review and pharmacologic risk reduction; yet improvements in salivary function are not consistently achieved through these interventions (Arany et al., 2021; Barbe, 2018). The limited effectiveness of these measures suggests that underlying genetic variation in drug metabolism may influence systemic anticholinergic burden and contribute to salivary dysfunction, providing a rationale for examining pharmacogenetic mechanisms. Genetic polymorphisms in genes encoding drug-metabolizing cytochrome enzymes, such as CYP2D6 and CYP2C19, significantly influence the pharmacokinetics of medications and their serum levels in the blood (Dong et al., 2018; Samer et al., 2013). Recent studies have reported a higher prevalence of anticholinergic side effects in patients carrying inactive genetic variants of *CYP2D6* and *CYP2C19*, responsible for the metabolic clearance of anticholinergic medications in the liver (Larsen and Rasmussen, 2017). While inherited variations in the metabolizing profile of genes encoding CYP enzymes may affect salivary secretion, only one study has previously explored the relationship between metabolizing profiles and xerostomia (Kersten et al., 2014). However, it did not comprehensively examine salivary outcomes across ADS, SAA, and objective salivary flow measures.

In this study, single nucleotide polymorphisms polymorphisms (SNPs) in *CYP2D6* and *CYP2C19* are associated with anticholinergic burden, as measured by ADS and SAA levels, as well as with saliva flow rate, in adults taking anticholinergic medications (Sevilla-Sanchez et al., 2018; Rhee et al., 2018). Specifically, we assessed the metabolizing phenotypes of two cytochrome enzymes, CYP2D6 and CYP2C19, which metabolize 50% of the most commonly used anticholinergic medications (Dong et al., 2018). We also explored whether the amount of saliva secreted from minor salivary glands exclusively under parasympathetic control is associated with anticholinergic burden (Boros et al., 1999; Bretz et al., 2000; Shruthi et al., 2021; Villa et al., 2016). We hypothesized that genetic variations in the *CYP2D6* and *CYP2C19* genes contribute to elevated anticholinergic burden and reduced saliva secretion. This was examined through associations between genetic variants, enzyme activity scores, and salivary outcomes, rather than predefined comparisons of metabolizer groups.

## Materials and methods

This prospective clinical study was approved by the University of Rochester Medical Center Institutional Review Board (RSRB #5666). *A priori* power estimation with a sample size of 93 participants indicated >80% power to detect medium effect sizes (Cohen’s *d* ≈ 0.5) at α = 0.05. This sample was therefore considered sufficient for testing the primary hypotheses regarding genotype associations. Participants (n = 99) were recruited and enrolled from the General Dentistry Specialty Care Clinic at the Eastman Institute for Oral Health. The middle-aged range (45–64 years) was chosen to minimize age-related variability in drug metabolism and salivary gland function (Affoo et al., 2015; Ziad et al., 2018). Informed consent was obtained from all individuals who met the eligibility criteria and agreed to participate in genetic testing. Participants were eligible for inclusion if they reported experiencing oral dryness (xerostomia) and had been taking at least one anticholinergic medication that is a confirmed substrate of CYP2D6 or CYP2C19 enzymes (minimum of 30 days prior to the study visit). Medication use was confirmed through a current, verified medication list and electronic health records. Individuals who had a diagnosis of Sjögren’s syndrome or any other systemic condition known to affect salivary gland function, a history of head and neck radiation or radioiodine treatment, documented liver disease or hepatic injury, or who were currently being treated with cholinergic agonists were not eligible. To reduce drug-drug interactions, individuals prescribed strong CYP2D6 or CYP2C19 inhibitors were excluded. Participants with diabetes were also excluded due to the independent effects of diabetes on salivary gland function, which could confound associations between anticholinergic burden, CYP450 phenotype, and salivary outcomes.

### Genotyping and allele definition

Participants underwent venous phlebotomy to collect whole blood (∼18.5 mL). All specimen aliquots were deidentified. Genomic DNA was purified using a commercial blood DNA mini kit from Qiagen (Germantown, MD) following the manufacturer’s instructions. Extracted DNA was quality-controlled for physical characteristics (concentration and purity) by Nano-Drop One spectroscopy (Thermo Scientific, Waltham, MA). DNA quality and amplifiability were also assessed by amplifying the beta-globin gene. Any specimen that failed quality control was re-extracted. Single-nucleotide polymorphism (SNP) genotyping was performed using the TaqMan™ Genotyper Software (Applied Biosystems, Foster City, CA). Quality control procedures included visual inspection of allele clusters and removal of samples with ambiguous calls. CYP2D6 and CYP2C19 star alleles were defined using the AlleleTyper™ Software (Thermo Fisher Scientific), allowing determination of functional diplotype, which informed enzyme activity scoring used in downstream variant-level analyses. The SNPs analyzed included 10 variants of the *CYP2D6* gene (*2, 3, 4, 6, 8, 10, 12, 14, 17, 41) and eight variants of the *CYP2C19* gene (*2, 3, 4, 5, 6, 7, 8, 17) (ClinPGx, 2025). A binary genetic-exposure risk variable was defined as the presence of at least one risk allele in selected *CYP2D6 or CYP2C19* variants combined with a relevant clinical criterion.

### Saliva flow assessments

Salivary function was assessed using two objective measures: unstimulated whole saliva (UWS) and minor saliva flow (MSF). To minimize circadian variability in salivary output, all collections were scheduled between 9:00 a.m. and 12:00 p.m. Participants were instructed to refrain from eating, drinking, smoking, brushing their teeth, using mouthwash, chewing gum, or rinsing their mouths for at least 1 hour prior to their appointment. UWS was collected over 10 min using a passive drooling method into pre-weighed plastic tubes while seated upright (Eliasson et al., 2009; Sreebny and Schwartz, 1997). Flow rates were recorded in milliliters per minute (mL/min). MSF from the upper lip was measured using an absorption technique. Cotton rolls were placed over the parotid duct openings to prevent contamination, and a standardized paper strip (5 mm × 35 mm) was positioned under the upper lip for 1 minute. Participants were asked to remain still to avoid mechanically induced salivary stimulation (Boros et al., 1999; Falcao et al., 2014). The flow rate was calculated based on the weight change of the strip, normalized to surface area and time, and reported in microliters per square centimeter per minute (µL/cm^2^/min). Severe hyposalivation was defined as UWS ≤0.1 mL/min, consistent with clinical criteria (Eliasson et al., 2009; Navazesh et al., 2008).

### Classification of study variables

The Anticholinergic drug scale (Khatri et al., 2022) was used to quantify each participant’s cumulative anticholinergic burden based on their current medication list (Carnahan et al., 2006; Sevilla-Sanchez et al., 2018). This score was derived from medications verified during the study visit and was modeled as an explanatory variable. Serum Anticholinergic Activity (SAA) was measured from blood samples collected in serum separator tubes. After centrifugation, serum was stored at −80 °C and analyzed using a radioreceptor assay that quantifies binding to muscarinic receptors (Nobrega et al., 2017). SAA values were expressed in atropine equivalents and used as either explanatory or intermediate variables, depending on the model. For categorical analyses, “high” SAA was defined *a priori* as values at or above the 90th percentile of the study distribution. Xerostomia severity was evaluated using an 11-item questionnaire, the Xerostomia Inventory (XI), with total scores ranging from 11 to 55 (Thomson et al., 1999). Higher scores indicate more severe subjective oral dryness. Covariates included age, biological sex, race, smoking status, and body mass index (BMI), all of which were recorded at the study visit and included in the adjusted analyses to control for potential confounding.

### Statistical analysis

Statistical analyses were performed using JMP® Pro 18.0.2, Microsoft Windows 10 Enterprise (10.0.19045.0) software. The normality of data was tested using the goodness-of-fit test. In statistical modeling, ADS and SAA were treated as explanatory or intermediate variables depending on the analysis. UWS, MSF, and XI scores were considered outcome variables. Age, biological sex, race, smoking status, and BMI were included as covariates to control for potential confounding effects. Normally distributed data were analyzed using ANOVA, followed by the Least Squares Means Student's t-test as a *post hoc* test. Normally distributed data were presented as mean ± SEM. Group differences were primarily assessed using the Mann–Whitney U and Wilcoxon/Kruskal–Wallis (Sums Rank) nonparametric tests, and the results were presented as medians ± interquartile ranges (IQRs). Effect sizes were calculated (Cliff’s delta for non-parametric, Cohen’s d for parametric tests) to assess the magnitude of group differences. Genotype Relative Risk (GRR) for single nucleotide polymorphisms (SNPs) significantly associated with a trait of interest was calculated as (probability of being a case given presence of risk genotype) divided by (probability of case given presence of low-risk genotype). Significance level alpha <0.05 was considered statistically significant.

## Results

Sociodemographic characteristics of the study sample are presented in Table 1. Biological sex differences were observed in several of the clinical variables assessed (Table 2). Women reported significantly more severe xerostomia symptoms compared to men, as indicated by a higher XI score (*p* = 0.021) as well as significantly lower unstimulated whole saliva (UWS) flow rates (*p* = 0.023), consistent with a greater subjective and objective dry mouth burden. Minor saliva flow (MSF) was also lower in women than in men (*p* = 0.044). Effect sizes for sex differences were small to moderate for MSF (Cliff’s delta = −0.26) and UWS (Cliff’s delta = −0.28).

**TABLE 1:** Sociodemographic characteristics of the study sample, including biological sex, race, smoking status, and age group.

Variable	n	Percent (%)
Biological sex		
Female	72	72.7
Male	27	27.3
Race		
European (EUR)	77	77.8
African American/Afro-Caribbean (Mossel et al., 2022)	9	9.1
American (AME)	7	7.1
Other	6	6.1
Smoking		
Never	48	48.5
Former	36	36.4
Yes, current	15	15.2

**TABLE 2:** Comparison of clinical and physiological characteristics by biological sex.

Variable	Female	Male	p-value	Test
ADS	3.0 [1.0, 4.5]	3.0 [1.0, 4.5]	0.5626	Mann–Whitney U
Age	56.0 [50.75, 60.0]	57.0 [51.5, 60.5]	0.9216	Mann–Whitney U
BMI	30.81 ± 7.79	31.60 ± 6.91	0.6013	t-test, mean ± SD
MSF (µL/cm^2^/min)	5.0 [3.5, 8.0]	6.5 [5.0, 8.5]	**0.0439**	Mann–Whitney U
SAA (pmol/mL)	0.16 [0.0, 1.06]	0.21 [0.0, 0.56]	0.6516	Mann–Whitney U
XI	37.0 [32.0, 40.25]	33.0 [25.0, 37.0]	**0.0205**	Mann–Whitney U
UWS (mL/min)	0.16 [0.07, 0.3]	0.25 [0.14, 0.51]	**0.0229**	Mann–Whitney U

Unless otherwise specified, values are presented as median and interquartile range [IQR].

ADS, anticholinergic drug score; BMI, body mass index; MSF, minor saliva flow; SAA = serum anticholinergic activity; XI, xerostomia index; UWS, unstimulated whole saliva flow.

The authors declare that the research was conducted in the absence of any commercial or financial relationships that could be construed as a potential conflict of interest. Values in bold are statistically significant (p < 0.05).

**FIGURE 1:**
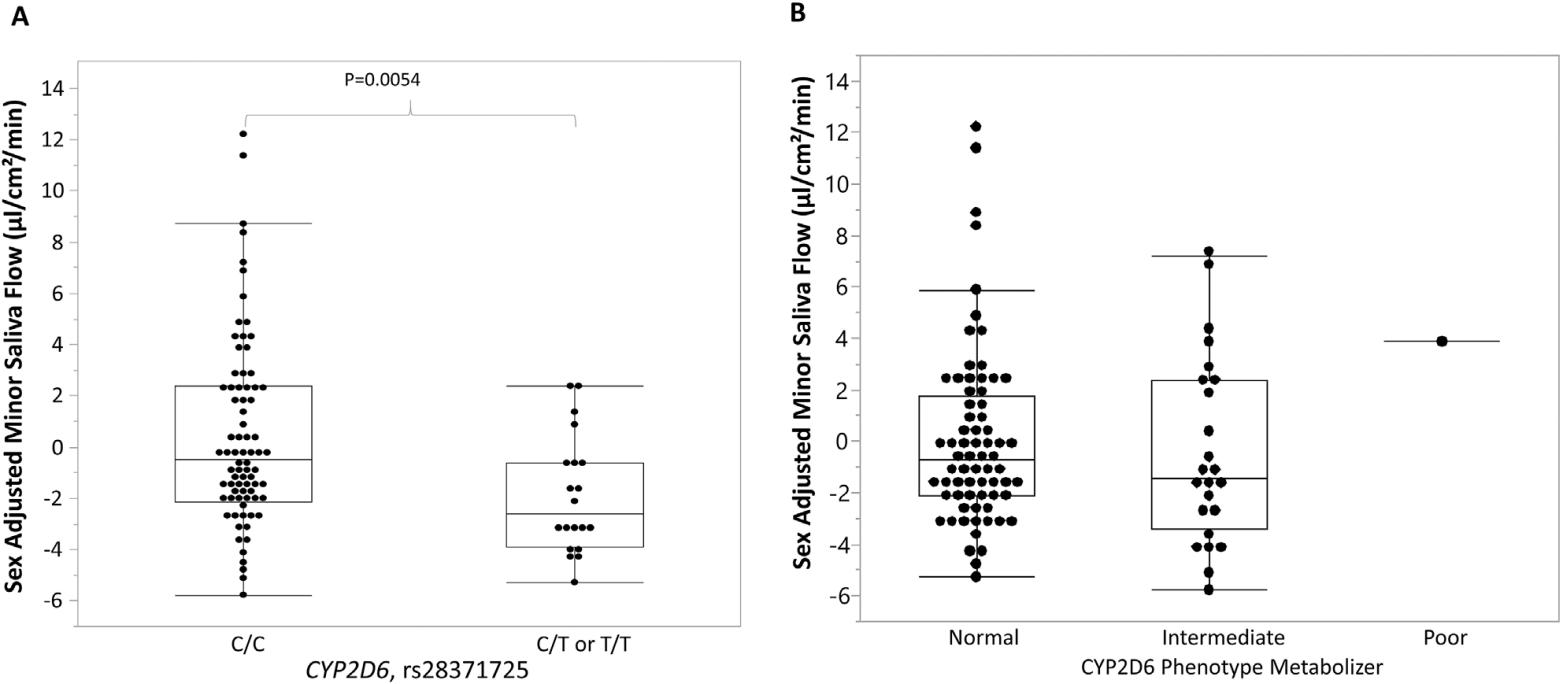
Association between rs28371725, CYP2D6 phenotype metabolizer, and sex-adjusted minor saliva flow (MSF). **(A)** Box-and-whisker plots show the distribution of minor saliva flow (MSF) values centered by sex, stratified by rs28371725 genotype under a dominant model (C/C = homozygous reference, C/T or T/T = carriers of the minor allele). Individuals carrying the minor allele (C/T or T/T) exhibited lower sex-adjusted MSF than those with the reference genotype (C/C). **(B)** Box-and-whisker plots show the distribution of MSF values centered by sex, stratified by the CYP2D6 phenotype metabolizer. No significant association was found between sex-adjusted MSF and CYP2D6 phenotypes. Boxes represent the interquartile range (IQR) with the horizontal line indicating the median, whiskers extend to 1.5 × IQR, and individual points represent observed values.

Therefore, in further analysis, sex adjusted MSF was applied. We found that MSF is significantly associated with a polymorphism in the *CYP2D6* gene (rs28371725, χ^2^ = 8.35, p = 0.0154). Individuals carrying at least one risk allele (C/T or T/T genotypes) had significantly lower MSF (−1.91 ± 2.30) compared to those carrying two low-risk alleles (C/C genotype) (0.38 ± 3.65, p-value = 0.0054) with a moderate effect size (Cliff’s delta = −0.39) (Figure 1A). Analyses of CYP2D6 phenotypes (normal, intermediate, and poor) revealed no significant associations with sex adjusted MSF (Figure 1B). Due to the limited number of SNPs genotyped, metabolizer status could not be confidently assigned for all participants. Eighty-eight participants were classified as normal, eight as poor metabolizers, and three were unresolved due to incomplete data. The small number of poor metabolizers limited statistical comparisons across metabolizer groups across the full range of CYP2D6 functional categories (poor, intermediate, normal, ultrarapid). Furthermore, no significant associations were identified between sex adjusted MSF and selected variants in *CYP2C19*.

**FIGURE 2:**
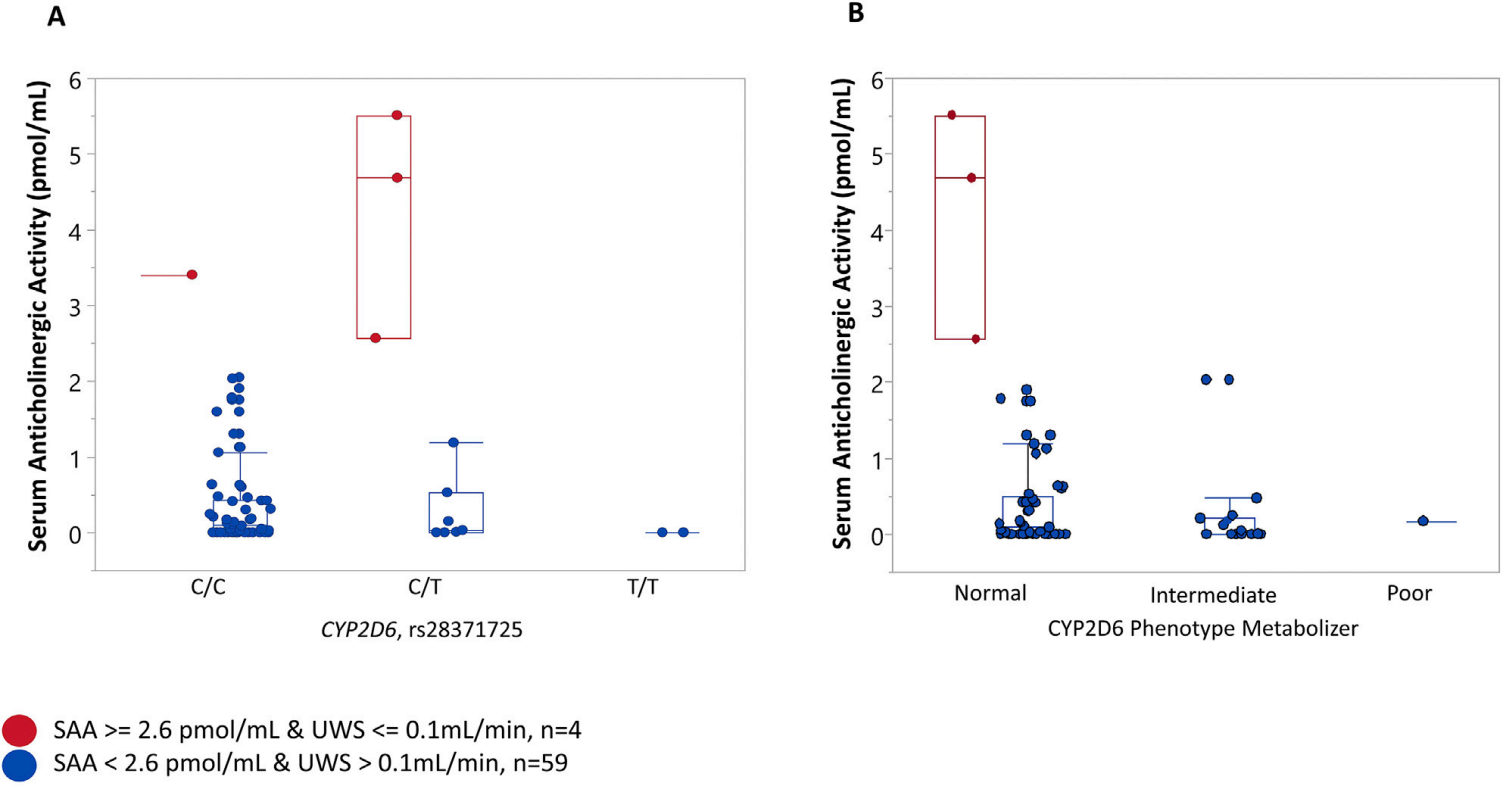
Association between a variant in *CYP2D6*, CYP2D6 phenotype metabolizer, and serum anticholinergic activity (SAA). Participants were stratified into “cases” when they presented with both “High” serum anticholinergic activity (SAA) (SAA ≥ 2.564 pmol/mL, 90th percentile) and dry mouth (unstimulated whole saliva flow (UWS) <= 0.1 mL/min) and “controls” when SAA levels were less than 2.564 and UWS >0.1 mL/min. **(A)** The majority of “controls” (84.75%) had the C/C genotype, 11.86% controls had the C/T genotype, and 3.39% were homozygous for the risk allele (T//T) genotype. Most cases (75%) were heterozygous for a rare allele (C/T genotype), and 25% of cases were homozygous for a common C allele (C/C) genotype. **(B)** No significant association was found between the CYP2D6 phenotype metabolizer and SAA. Three “cases” (one of the cases could not have a metabolizer phenotype resolved) had a normal CYP2D6 metabolizer phenotype. Most “controls” (72%) had a normal, 26% had intermediate, and 0.02% (one individual) had a poor CYP2D6 metabolizer phenotype.

To further explore pharmacogenetic influences, we investigated the association of selected single nucleotide polymorphisms (SNPs) in the *CYP2D6* or *CYP2C19* genes with serum anticholinergic activity (SAA). Given the absence of a universal clinical cutoff, the 90th percentile of SAA (Lopez-Alvarez et al., 2019) in our study population was used as a cutoff point to identify individuals with potentially high levels. In our study sample, participants with SAA ≥ 2.564 pmol/mL (90th percentile of SAA) were considered to have “high” SAA levels (n = 10). Participants were further stratified based on their UWS flow into those with severe hyposalivation (also known as dry mouth) (UWS <0.1 mL/min, n = 31). To assess whether genetic variations in *CYP2D6* and *CYP2C19* were associated with SAA and dry mouth, we compared genotype frequencies of those who had high SAA and dry mouth (n = 4) to those who had no high SAA and no dry mouth (n = 59). A single nucleotide polymorphism in the *CYP2D6* gene (rs28371725) was significantly associated with SAA levels and dry mouth status (χ2 = 7.734, p-value = 0.0209) with a large effect size (Φ = 0.30) in Figure 2A. The majority (84.75%) of study participants had no high SAA level (SAA <2.564 pmol/mL) and no dry mouth (UWS >0.1 mL/min), with the low-risk genotype. Genotype relative risk (GRR) for rs28371725 under the dominant model was calculated as (probability of being a case given 1 or 2 risk alleles)/(probability of being a case given zero risk alleles) and was estimated to be 12.75 (95% CI: 1.45, 112.12). No significant associations were identified between CYP2D6 metabolizer phenotypes and cases defined as individuals having SAA ≥ 2.564 pmol/mL and dry mouth (UWS <0.1 mL/min) (Figure 2B). Furthermore, no significant associations were identified between selected variants in *CYP2C19* and SAA and dry mouth.

**FIGURE 3:**
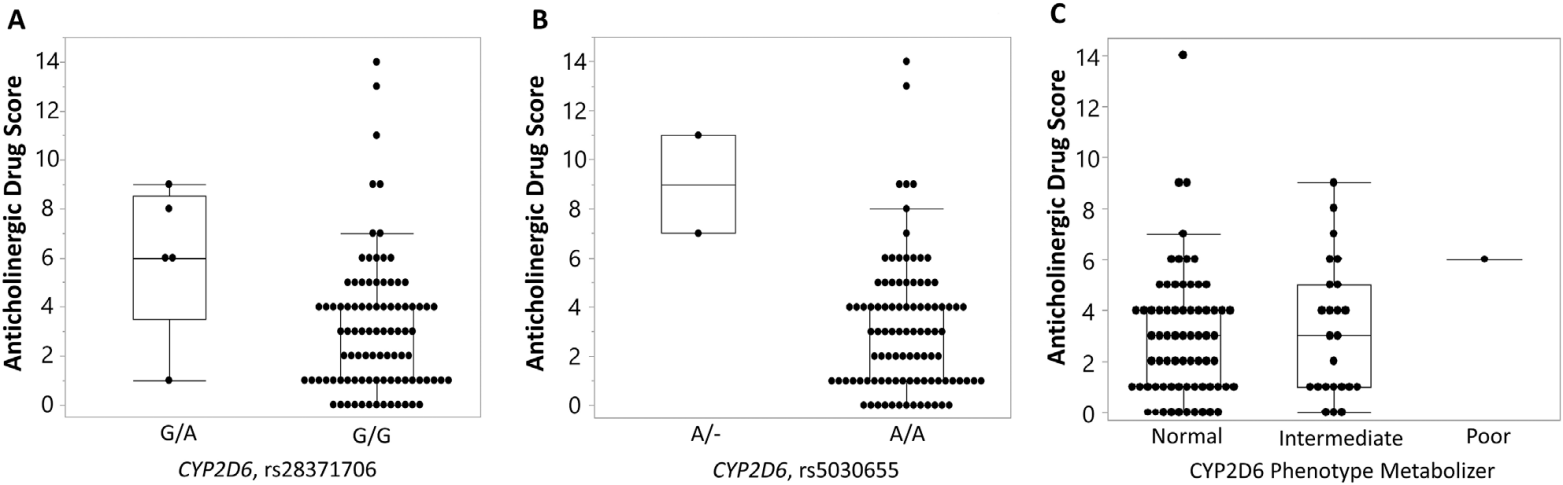
Association of *CYP2D6* and phenotype metabolizer with variants with anticholinergic drug scale (ADS) (Khatri et al., 2022). Box-and-whisker plots display anticholinergic drug scale (ADS) values stratified by genotype at two *CYP2D6* variants: rs28371706 **(A)** and rs5030655 (single base deletion) **(B)**, and a CYP2D6 phenotype metabolizer **(C)**. For rs28371706, individuals carrying the G/A genotype exhibited higher ADS compared with those homozygous for the reference G/G genotype. For rs5030655, carriers of the single base deletion allele (A/–) showed elevated ADS relative to A/A homozygotes. No significant association was found between ADS values and CYP2D6 phenotypes. Boxes represent the interquartile range (IQR) with the horizontal line denoting the median, whiskers extend to 1.5 × IQR, and individual points indicate observed values.

**FIGURE 4:**
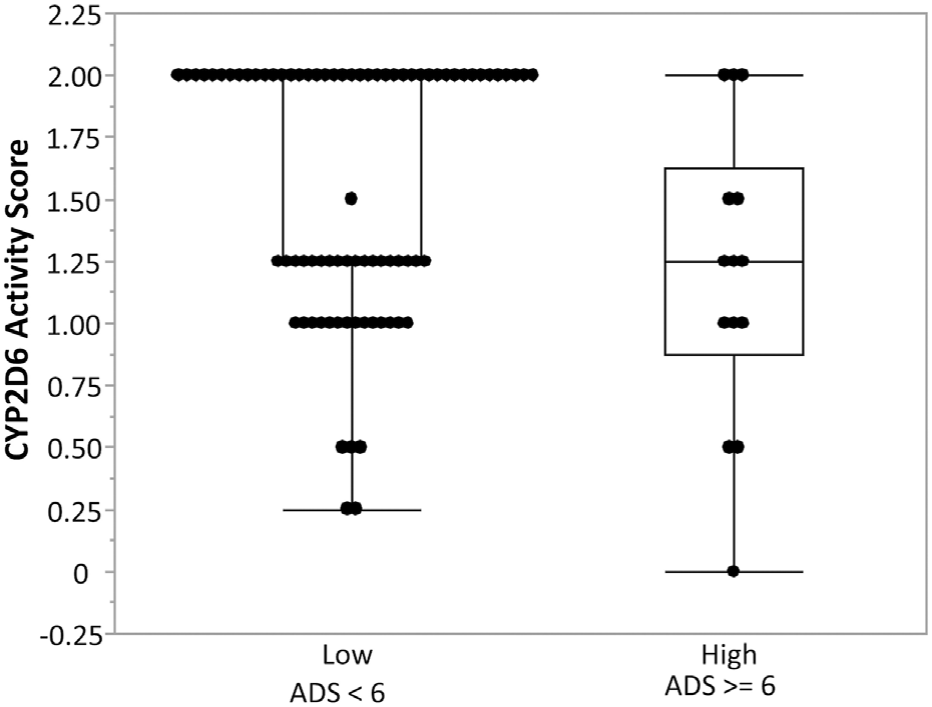
Association between CYP2D6 enzyme activity score and anticholinergic drug scale (ADS) (Khatri et al., 2022). Box-and-whisker plots illustrate CYP2D6 enzyme activity scores stratified by anticholinergic drug scale (ADS) categories: low ADS (<6) and high ADS (≥6). Individuals with high ADS demonstrated lower CYP2D6 enzyme activity compared to those with low ADS, suggesting reduced CYP2D6 metabolic capacity is associated with greater anticholinergic burden. Boxes represent the interquartile range (IQR) with the horizontal line denoting the median, whiskers extend to 1.5 × IQR, and individual points represent observed values.

We also evaluated an association between selected SNPs and the anticholinergic drug scale (ADS) (Khatri et al., 2022). Two variants in the *CYP2D6* gene (rs28371706 and rs5030655) were significantly associated with ADS (p-value = 0.0139 and p-value = 0.0019, respectively). Individuals heterozygous for a risk allele (A) (G/A genotype) at rs28371706 had significantly higher average ADS (6.0 ± 3.08) compared to those with zero copies of a risk allele (G/G genotype) (3.09 ± 2.75, p-value = 0.0139) with a large effect size (Cliff’s delta = 0.48) (Figure 3A). Similarly, individuals heterozygous for a single base deletion (A/- genotype) at rs5030655 had significantly higher average ADS (9.0 ± 2.83) compared to those homozygous for a low-risk allele (normal function, A/A genotype) (3.11 ± 2.71, p-value = 0.0019) with a large effect size (Cliff’s delta = 0.59) (Figure 3B). No significant associations were identified between CYP2D6 metabolizer phenotypes and ADS (Figure 3C). To calculate genotype relative risk for ADS, participants were divided into “high” anticholinergic burden ADS (ADS ≥ 6) and “low” anticholinergic burden (ADS <6). Genotype relative risk (the probability of “high anticholinergic activity” given risk genotype)/(the probability of “high anticholinergic activity” given low risk genotype) for the two variants, rs28371706 and single base deletion (rs5030655), was estimated to be 6.2 (95% CI: 3.12, 12.3) and 6.86 (95% CI: 4.22, 11.13), respectively. Additionally, ADS was significantly associated with the CYP2D6 enzyme activity score (p-value = 0.0358). The median CYP2D6 activity score was significantly higher among individuals with low anticholinergic activity (2, IQR = 0.75) compared to those with high anticholinergic activity (1.25, IQR = 0.75, p-value = 0.0358) with a moderate effect size (Cliff’s delta = −0.35) (Figure 4). No significant associations were identified between *CYP2C19* variants and ADS.

## Discussion

In this study, we investigated the associations between genetic polymorphisms in *CYP2D6* and *CYP2C19* genes, minor saliva flow (MSF), serum anticholinergic activity (SAA), and anticholinergic drug score (Khatri et al., 2022). We found that genetic variations in *CYP2D6* are consistently associated with MSF, SAA, and ADS among study participants. This pharmacogenetic study extends earlier findings from our prior investigation (Michail et al., 2023), which identified associations between anticholinergic medication use and reduced salivary flow. We explored through CYP450 genotyping whether individual differences in drug metabolism help explain the observed variability in both anticholinergic burden and salivary outcomes. In our study, *CYP2D6* variants were associated with elevated SAA and reduced salivary flow, helping to clarify the inconsistent relationships between ADS and SAA reported in earlier studies. Lampela and colleagues (Lampela et al., 2017) demonstrated that ADS reflects cumulative drug exposure, whereas SAA indicates biologic receptor blockade; however, the two measures often correlate poorly. Kersten and co-workers (Kersten et al., 2013) also reported discrepancies between ADS and SAA, and Hori et al. (2014) observed that SAA does not always align with drug-based scales. Our findings suggest that pharmacogenetic differences in CYP activity may account for part of this divergence, providing a mechanistic link between drug exposure, systemic anticholinergic activity, and clinical outcomes.

The current study adjusted for sex differences in MSF, confirming that genetic polymorphism in *CYP2D6* (rs28371725) is significantly associated with sex-adjusted MSF. Participants carrying at least one risk allele had significantly lower MSF compared to those carrying two low-risk alleles. This observation highlights the potential impact of *CYP2D6* genetic variation on salivary gland function, likely through altered metabolism of medications with anticholinergic properties. Our adjustment for sex is consistent with prior evidence that minor salivary gland secretion differs by biological sex. Eliasson and Carlén (Eliasson and Carlen, 2010) described both age- and sex-related variations in minor salivary gland secretion, (Falcao et al., 2014) established reference values showing that women generally have lower labial gland output, and Villa and coworkers (Villa et al., 2016) confirmed that female sex predicts reduced labial secretion in population-based cohorts. Importantly, several studies emphasize that impairment of minor glands, despite their lower contribution to overall salivary volume, has a disproportionately large impact on the sensation of oral dryness. The *CYP2D6* association observed here, therefore, adds a genetic dimension to glandular function, which is already known to be influenced by biological sex and is particularly relevant to hyposalivation.

Furthermore, the association between rs28371725 in *CYP2D6* and the combination of high SAA and dry mouth status suggests a possible gene-environment interaction. In this study, a data-driven approach was used to define “high SAA” as values at or above the 90th percentile of the cohort distribution (≥2.564 pmol/mL). This threshold identifies individuals with relatively elevated systemic anticholinergic activity, consistent with prior literature recommending percentile-based cutoffs when standardized reference ranges are lacking. Notably, our empirically derived cutoff for high SAA (≥2.564 pmol/mL; 90th percentile) closely aligns with the gold-standard threshold of ≥2.80 pmol/mL reported by Mulsant et al. (2004) in a population-based study. Given that SAA measurement requires technically demanding radioreceptor assays and is therefore rarely performed, this similarity reinforces the validity of our data-driven threshold and highlights the clinical relevance of our findings. The genotype relative risk (GRR) estimates of 12.75 under the dominant model indicate that individuals carrying one or more risk alleles are at a substantially higher risk of experiencing high SAA and dry mouth. This result supports the hypothesis that CYP2D6-mediated metabolism of anticholinergic drugs influences systemic anticholinergic activity and its downstream effects on salivary flow.

Two other variants in *CYP2D6* (rs28371706 and single base deletion rs5030655) were significantly associated with ADS, a widely used measure of cumulative exposure to anticholinergic medications. These findings align with previous reports relating *CYP2D6* polymorphisms to variations in drug metabolism, particularly for medications with anticholinergic properties (Samer et al., 2013; Trenaman et al., 2021; Zhou et al., 2017). Additionally, we observed an inverse association between the CYP2D6 enzyme activity score and ADS, indicating that reduced metabolic activity is associated with a greater anticholinergic burden. Our findings build on prior work evaluating pharmacogenetic influences on anticholinergic burden. An earlier study by Kersten et al. (2014) carefully examined the relationship between CYP2D6 and CYP2C19 metabolizer status and systemic anticholinergic activity but did not observe strong or consistent associations. Our analysis extends this work by demonstrating significant associations between specific *CYP2D6* variants, elevated SAA, and reduced salivary flow. Differences in study design and population may account for the contrasting results: the earlier cohort included predominantly older adults with multiple comorbidities, whereas our sample consisted of middle-aged individuals with fewer confounding factors, which may have allowed genetic effects to emerge more clearly.

By including both ADS and SAA, our study assessed whether the CYP450 metabolizer phenotype accounts for the variation between medication-based and biologic measures of anticholinergic activity. Our findings suggest that *CYP2D6* variation may contribute to interindividual variability in anticholinergic burden and hyposalivation risk, in part through differences in metabolic clearance. Individuals with reduced CYP2D6 enzyme activity have diminished enzymatic function, leading to slower metabolic clearance, increased systemic exposure to anticholinergic drugs, and consequently higher ADS scores. This exposure-driven interpretation is supported by consistent associations in our data, including higher serum anticholinergic activity and lower salivary flow in participants with lower CYP2D6 enzyme activity scores. CYP2D6 metabolizes a broad range of medications—accounting for nearly one-quarter of all drugs in clinical use, including many anticholinergic substrates, making the gene encoding it (*CYP2D6*) a critical pharmacogene in precision therapeutics (Taylor et al., 2020). Individuals carrying reduced-function or nonfunctional *CYP2D6* alleles may accumulate increased levels of anticholinergic burden and be predisposed to hyposalivation. However, genotype alone does not always predict enzyme activity due to phenoconversion (i.e., changes in metabolic function by comedications) (Nahid and Johnson, 2022). Our findings suggest that *CYP2D6* genotypes may guide clinicians in selecting anticholinergics less dependent on CYP2D6 metabolism, using lower starting doses, or increasing monitoring for adverse effects in genetically susceptible individuals. Additionally, integrating *CYP2D6* genotype data into electronic prescribing systems or clinical decision-support tools could enable more personalized, safer anticholinergic regimens. The preemptive pharmacogenetic testing trials (e.g., The PREPARE trial and other clinical implementations) demonstrate that genotype-guided prescribing can reduce adverse drug reaction rates in other therapeutic areas (Chenchula et al., 2024). Translation of our findings into clinical practice would require prospective trials including larger cohorts that would evaluate not only salivary outcomes but also medication efficacy, adherence, and the cost-benefit of genotyping in populations at risk for polypharmacy and xerostomia.

This study has several limitations, including the cross-sectional design, which precludes causal inferences, the modest sample size, and the lack of functional validation of the identified SNPs. The achieved sample size of 99 limited the power for genetic associations involving rare alleles or small effect sizes. Consequently, findings for infrequent genotypes and extreme outcome subgroups should be considered exploratory and interpreted cautiously. Only ten SNPs within the *CYP2D6* gene were genotyped, preventing comprehensive coverage of structural variants required for accurate diplotype-based metabolizer categorization. Consequently, metabolizer status could not be confidently assigned for all participants: eighty-eight were classified as normal, eight as poor metabolizers, and three were unresolved due to incomplete data. The small number of poor metabolizers limited statistical comparisons across metabolizer groups across the full range of CYP2D6 functional categories (poor, intermediate, normal, ultrarapid). Analyses were instead conducted at the level of individual variants and enzyme activity scores, which offer finer resolution in small samples but reduce comparability with standard clinical guidelines based on categorical phenotypes. Future work should integrate both approaches to fully characterize pharmacogenetic risk. Our report presents baseline findings from a prospective longitudinal study, focused on salivary outcomes and related covariates that have been consistently associated with anticholinergic burden in previous cross-sectional studies. Other oral and dental health conditions, such as caries incidence and periodontal status, will be analyzed in future follow-up, as their multifactorial etiology is best addressed within a longitudinal framework. Additional limitations include heterogeneity in the types of anticholinergic medications used and reliance on a single clinical cohort of middle-aged adults, which may limit the generalizability of the findings. Finally, without direct measurements of drug plasma concentrations or functional enzyme assays, the observed associations between *CYP2D6* polymorphisms and anticholinergic burden remain indirect. Future research should incorporate longitudinal designs and functional studies to elucidate the molecular mechanisms underlying these associations.

Although we identified specific variants in *CYP2D6* that were significantly associated with anticholinergic burden and reduced salivary secretion, these pharmacogenetic variants are insufficient to fully explain the variability in hyposalivation. Some participants with severe hyposalivation lacked *CYP2D6* or *CYP2C19* risk alleles, while others with multiple risk alleles maintained preserved secretion. This suggests the likely involvement of additional genetic factors, possibly in pathways related to autonomic innervation, glandular secretion, or immune modulation, which warrant further investigation. Overall, these results suggest that genetic variation in *CYP2D6* contributes to interindividual differences in anticholinergic drug metabolism, systemic anticholinergic burden, and hyposalivation. These findings have important implications for personalized medicine, particularly in managing polypharmacy and its associated adverse effects. Future studies with larger sample sizes and diverse populations are needed to validate these associations and explore the underlying mechanisms linking *CYP2D6* genetic variation, anticholinergic activity, and salivary gland dysfunction.

In conclusion, our findings emphasize the importance of considering genetic variability in *CYP2D6* when evaluating the risk of anticholinergic-related side effects, such as hyposalivation. Pharmacogenetic testing may be valuable for identifying individuals at heightened risk of these adverse effects, ultimately enabling more personalized and safer medication regimens. This direction expands on current clinical understanding that anticholinergic-induced hypofunction varies significantly across medications, reflecting differences in muscarinic receptor binding affinity and reported risk ratios (Arany et al., 2021). Recognizing and substituting high-risk medications, when medically feasible, remains a practical strategy for mitigating anticholinergic side effects. Pharmacogenetic profiling may add another layer of individualization by identifying patients with *CYP2D6* or *CYP2C19* risk alleles, who may be more prone to systemic accumulation and salivary dysfunction. Integrating pharmacogenetic information with existing knowledge on drug-specific salivary risk may offer a more individualized framework for anticipating hyposalivation, particularly in patients with polypharmacy or underlying risk factors. While still exploratory, this approach is consistent with the broader aim of advancing precision medicine in oral health.

## Data Availability

The original contributions presented in the study are included in the article/Supplementary Material, further inquiries can be directed to the corresponding author.
